# The neural crest‐associated gene ERRFI1 is involved in melanoma progression and resistance toward targeted therapy

**DOI:** 10.1002/1878-0261.70137

**Published:** 2025-10-03

**Authors:** Nina Wang, Qian Sun, Daniel Novak, Lei Zhu, Juliane Poelchen, Tamara Steinfass, Yiman Wang, Viktor Umansky, Jochen Utikal

**Affiliations:** ^1^ Skin Cancer Unit German Cancer Research Center (DKFZ) Heidelberg Germany; ^2^ Department of Dermatology, Venereology and Allergology, University Medical Center Mannheim Ruprecht Karl University of Heidelberg Mannheim Germany; ^3^ DKFZ‐Hector Cancer Institute at the University Medical Center Mannheim Germany; ^4^ JCCU Translational Surgical Oncology (A430) German Cancer Research Center (DKFZ) Heidelberg Germany; ^5^ Department of Surgery, University Medical Center Mannheim, Medical Faculty Mannheim Heidelberg University Mannheim Germany; ^6^ Mannheim Institute for Innate Immunoscience (MI3), Medical Faculty Mannheim University of Heidelberg Mannheim Germany

**Keywords:** BRAFi, drug resistance, ERRFI1, melanoma, miR‐200c, NC

## Abstract

Targeted therapy has been established as a therapeutic option for the treatment of metastatic melanoma. Despite initially being very efficient, many tumors develop resistance to targeted therapy, leading to its failure. We previously demonstrated that the neural crest (NC)‐associated gene ERRFI1 is highly expressed in metastatic melanoma and correlates with a bad prognosis. Here, we show that the expression of ERRFI1 was upregulated in melanoma and negatively correlated with the expression of melanocytic differentiation markers, such as MITF and TYR. Downregulation of ERRFI1 with the help of siRNA increased the susceptibility of melanoma cells toward BRAF inhibition (BRAFi) and resensitized BRAFi‐resistant melanoma cells to BRAFi. Mass spectrometry‐based proteomic analysis revealed that ERRFI1 silencing diminished the activation of the mitogen‐activated protein kinase (MAPK) and AKT signaling pathways, which usually contribute to drug resistance. Furthermore, we show that miR‐200c targeted the 3′UTR of ERRFI1 and reduced its expression, resulting in the resensitization of BRAFi‐resistant melanoma cells to BRAFi. Our study results suggest that ERRFI1 could be a potential therapeutic target for the treatment of metastatic melanoma.

## Graphical abstract

ERRFI1, a neural crest (NC)‐associated gene, was upregulated in melanoma and negatively correlated with the expression of melanocytic differentiation markers and the susceptibility of melanoma cells toward BRAF inhibitors (BRAFi). Knocking down ERRFI1 significantly increased the sensitivity of melanoma cells to BRAFi. Additionally, the miRNA miR‐200c was found to markedly reduce ERRFI1 expression and thereby resensitize melanoma cells to BRAFi.

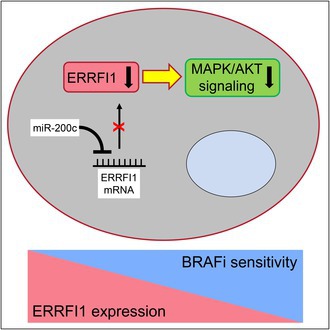

**Abbreviations**
18S: ribosomal RNA
AKT: v‐akt murine thymoma viral oncogene
ATCC: American Type Culture Collection
AXL: AXL receptor tyrosine kinase
BRAF: B‐RAF proto‐oncogene, serine/threonine kinase
BRAFi: BRAF inhibitors
BrdU: bromodeoxyuridine
DMSO: dimethylsulfoxide
ERK: extracellular signal‐regulated kinase
ERRFI1: ERBB receptor feedback inhibitor 1
FITC: fluorescein isothiocyanate
KD: knockdown
LC–MS/MS: liquid chromatography–tandem mass spectrometry
MAPK: mitogen‐activated protein kinase
miRNA: microRNAs
MITF: microphthalmia‐associated transcription factor
NC: neural crest
NHM: normal human melanocytes
PI: propidium iodide
PI3K: phosphatidylinositol‐4,5‐bisphosphate 3‐kinase
siRNA: small interfering RNA
SOX10: SRY‐box transcription factor 10
TMA: tissue microarray
TYR: tyrosinase
Vem: vemurafenib

## 1 Introduction

Melanoma, the most lethal form of skin cancer, originates from melanocytes, which stem from precursor cells from the neural crest (NC) [1]. This unique embryonic origin could explain the aggressive characteristics of melanoma. NC formation, cell migration, and melanocytic maturation are regulated by a network of transcription factors, such as PAX3, MSX1, SOX10, and MITF [2]. Numerous studies have demonstrated that the transcription factors involved in NC cell development and melanocyte formation are also expressed in melanoma and contribute to the plasticity of melanoma cells [2]. Moreover, the substantial intratumoral heterogeneity and cellular flexibility of melanoma add to its aggressive nature by enabling rapid adaptation to the tumor microenvironment and acquiring drug resistance [3, 4].

Notably, approximately 50% of melanomas exhibit a BRAF mutation, particularly BRAF V600E, which constitutively activates the BRAF protein and the downstream mitogen‐activated protein kinase (MAPK) pathway [5]. BRAF and MEK inhibitors (BRAFi and MEKi) have significantly enhanced treatment outcomes for patients with BRAF mutations, demonstrating excellent initial response [6]. However, their long‐term effectiveness is often limited by the development of acquired resistance, which leads to clinical relapse [7]. Research indicates that around 50% of patients develop acquired resistance within 1 year, and this figure rises to approximately 80% within 5 years [8, 9, 10]. The complexity of the mechanisms behind this drug resistance presents a major challenge in effectively combating it.

Our previous studies have identified the ERBB receptor feedback inhibitor 1 (ERRFI1) as an NC‐associated gene highly expressed in metastatic melanoma and associated with a poor prognosis [11]. While several studies have shown that ERRFI1 is linked to poorer clinical outcomes and drug resistance in various cancers, its association with melanoma drug resistance has not yet been explored [12, 13, 14, 15, 16, 17]. In this study, we aimed at investigating whether targeting ERRFI1 could serve as a strategy to combat melanoma drug resistance and enhance treatment effectiveness.

## 2 Materials and methods

### 2.1 Cell culture and development of vemurafenib‐resistant cell lines

Human melanoma cell lines HT144 (RRID: CVCL_0318) and SK‐MEL‐28 (RRID: CVCL_0526) were purchased from ATCC (Manassas, VA, USA). All original cell lines have been authenticated using STR (or SNP) profiling within the last 3 years by the company Multiplexion (Friedrichshafen, Germany). All cell lines used in this study have been tested negative for mycoplasma contamination with Venor®GeM Classic Mycoplasma Kit (Minerva Biolabs, Berlin, Germany). All cell lines were cultured in DMEM (Gibco, Schwerte, Germany) supplemented with 10% heat‐inactivated FBS (Sigma), 0.1 mm β‐mercaptoethanol (Gibco), 1% nonessential amino acids (NEAA; Sigma, Hamburg, Germany), and 1% penicillin/streptomycin (Sigma) in a humidified incubator at 5% CO_2_ and 37 °C.

To develop vemurafenib‐resistant melanoma cell lines, HT144 and SK‐MEL‐28 were exposed to gradually increasing concentrations of the BRAF V600E inhibitor Vem over 6 months, as described previously [18]. Additionally, vemurafenib‐resistant and parental WM9 cells were kindly provided by Dr. Ewelina Dratkiewicz of the University of Wroclaw, Poland [19].

### 2.2 Immunohistochemistry of tissue microarray (TMA)

Tumor samples from melanoma patients were utilized to prepare TMA slides. These slides were stained using an anti‐ERRFI1 antibody (HPA027206; Atlas Antibodies, Stockholm, Sweden) and scanned by the NCT Gewebebank facility at the pathology unit of the University of Heidelberg. TMAs were assessed using a scoring system based on the quantity and intensity of the staining [20]. The study methodologies conformed to the standards set by the Declaration of Helsinki. The experiments were undertaken with the understanding and written consent of each subject. The study methodologies were approved by the local ethics committee (ethics committee II of Heidelberg University, Germany) under the license number 2014‐835R‐MA.

### 2.3 Transfection of cells

Cells were seeded at approximately 60% confluency in 6‐well plates. The following day, cells were transfected with two different siRNA targets against ERRFI1 (siERRFI1‐1: HSS122815, Thermo Fisher Scientific, Waltham, MA, USA; siERRFI1‐2: SR310045AL, Origene, Herford, Germany) or a control siRNA (12935400; Thermo Fisher Scientific) using Lipofectamine RNAiMAX Transfection Reagent (13778‐150; Invitrogen, Erlangen, Germany), according to the manufacturer's protocol. Forty‐eight hours after transfection, RT‐qPCR and western blot assays were used to validate the transfection efficiency. miR‐200c mimics (339173; Qiagen, Hilden, Germany) and negative control 4 miRNA mimics (339173; Qiagen) were also transfected using the same reagent 56 h before conducting further experiments.

### 2.4 Quantitative real‐time PCR (qRT‐PCR)

RNA was extracted using the RNeasy Mini Kit (Qiagen) according to the manufacturer's instructions. For cDNA synthesis, 500 ng of RNA was used with the RevertAid First Strand cDNA Synthesis Kit. qRT‐PCR was performed using SYBR Green PCR Master Mix, following previously established protocols [21]. Primer sequences are listed below:

AXL: 5′ CCGTGGACCTACTCTGGCT 3′, 5′ CTTCGGGTATTGCGGTTCC 3′;
ERRFI1: 5′ GAGCAGTCGCAGTGAGTT 3′, 5′ GTGAACCCGTACGAAGGTT 3′;
GAPDH: 5′ GAAGGTGAAGGTCGGAGTC 3′, 5′ CTTTAGGGTAGTGGTAGAAG 3′;
SOX10: 5′ GGCTTTCTGTCTGGCTCACT 3′, 5′ GGGGGTCCTTACTGGGAGAT 3′;
18S: 5′ GAGGATGAGGTGGAACGTGT 3′, 5′ TCTGGACCTCGCTGACTTCT 3′.

For miRNA analysis, 10 ng of total RNA was reverse‐transcribed using the TaqMan microRNA Reverse Transcription Kit (4366596; Thermo Fisher Scientific) and analyzed with TaqMan MicroRNA Assays (4427975; Thermo Fisher Scientific).

### 2.5 Western blot analysis

Proteins were extracted using RIPA buffer and quantified with the bicinchoninic acid assay (BCA). Between 30 and 40 μg of protein were loaded per lane for western blot analysis using primary antibodies against ERRFI1 (HPA027206; ATLAS Antibodies), β‐actin (5125S; Cell Signaling, Frankfurt, Germany), α‐actinin (H‐2) (sc‐17829; Santa Cruz, Dallas, TX, USA), ERK (4695S; Cell Signaling), p‐ERK (9106S; Cell Signaling), AKT (2920S; Cell Signaling), and p‐AKT (4058S; Cell Signaling). Densitometric protein quantification was performed using the image lab (Bio‐Rad, Feldkirchen, Germany) software.

### 2.6 Bromodeoxyuridine (BrdU) enzyme‐linked immunosorbent assay (ELISA) proliferation assay

A total of 1–2 × 10^4^ cells (control or ERRFI1 knockdown) in 100 μL of culture medium were seeded per well of a 96‐well plate. The next day, 20 μL of 1× BrdU was added to each well and incubated for 6–24 h. The proliferation assay was conducted according to the manufacturer's protocol (ab126556; Abcam, Cambridge, Great Britain), and absorbance was measured at a dual wavelength of 450/550 nm using a Tecan (Männedorf, Switzerland) plate reader.

### 2.7 Colony formation assay

Between 1000 and 2000 cells were seeded per well of a 6‐well plate. The following day, Vem was added to a final concentration of 10 μm. The medium was changed after 24 h. After 10–14 days, cell colonies were stained with 0.5% crystal violet, and the number of colonies was quantified using the imagej (Bethesda, MD, USA) software.

### 2.8 Drug sensitivity assay

Melanoma cells were plated at a density of 3000–5000 cells per well of a 96‐well plate and allowed to adhere for 24 h. Cells were then treated with different concentrations of Vem, ranging from 0.0001 to 25 μm. Cell viability was assessed 48/72 h later using the alamarBlue cell viability assay (DAL1100; Invitrogen).

### 2.9 Cell apoptosis assay

Cells were seeded at approximately 60% confluency in 6‐well plates. The next day, Vem was added and maintained for 48 h followed by staining with fluorescein isothiocyanate (FITC) and propidium iodide (PI), according to the manufacturer's protocol (556547; BD Biosciences, Heidelberg, Germany). Samples were analyzed by flow cytometry at the DKFZ Core Facility, and the data were analyzed using the flowjo (Ashland, OR, USA) software.

### 2.10 Mass spectrometry

Melanoma cells (HT144, SK‐MEL‐28, and WM9) were transfected with ERRFI1 siRNA, and 48 h later, prepared for proteomic analysis. Proteins were extracted using RIPA buffer supplemented with 10 mm NaF, 1 mm Na_3_VO_4_, 1× Complete EDTA‐free protease inhibitor (4693159001; Merck), 1× PhosStop (4906845001; Merck), 250 U·mL^−1^ Benzonase (70746‐3; Merck, Darmstadt, Germany), and 10 U·mL^−1^ DNase (79256; Qiagen). Protein lysate was analyzed by liquid chromatography–tandem mass spectrometry (LC–MS/MS) at the DKFZ Genomic and Proteomics Core Facility.

### 2.11 miRNA conserved target site prediction

Putative miRNA target sites in the 3′ UTR of the ERRFI1 gene were identified using miRDB and TargetScan. The analysis focused on the 3′ UTR of the ERRFI1 transcript ENST00000377482.5 to find potential miRNA binding sites.

### 2.12 Public datasets

ERRFI1 expression levels in melanocytes and melanoma cells were analyzed from datasets from the GEO database (GSE130244 and GSE111766) and the cBioportal database (DFCI Science 2015), along with the mRNA expressions of AXL, SOX10, MITF, TYR, DCT, and MLANA. The expression levels of ERRFI1 in primary and metastatic melanoma were also assessed using data from The Cancer Genome Atlas (TCGA). Survival analysis was performed using data from the DFCI Science 2015 database (www.cbioportal.org).

### 2.13 Proteomic analysis

Database for Annotation, Visualization, and Integrated Discovery (DAVID) was used to conduct GO enrichment and KEGG pathway analysis. Gene set enrichment analysis was performed using GSEA. STRING database and cytoscape were used to build and visualize the PPI network for differentially expressed proteins.

### 2.14 Spheroid formation

Melanoma cells were seeded at approximately 60% confluency in 6‐well plates for siRNA transfection. After 48 h, fresh medium containing Nanoshuttle‐PL (657843; Greiner, Frickenhausen, Germany) was added to magnetize the cells. Subsequently, 25 000–50 000 cells were plated in a 96‐well round bottom ultra‐low attachment plate (7007; Corning, Corning, NY, USA), and a magnet plate was used to induce cell aggregation. Further experiments were performed after stable spheroid formation.

### 2.15 Dual‐reporter luciferase assay

For the luciferase reporter assay, HEK293T cells were seeded at a density of 10 000 cells per well in 96‐well plates and incubated for 24 h. Cells were then co‐transfected with either miR‐200c mimics or miR‐NC mimics, together with the control or ERRFI1 3′ UTR plasmid, which was cloned into the pmirGLO Dual‐Luciferase vector (Promega), using Lipofectamine 3000 (L3000015; Thermo Fisher Scientific). Each condition was performed in triplicate. After 48 h of transfection, firefly and Renilla luciferase activities were measured with the Dual‐Luciferase Reporter Assay System (E2920; Promega, Walldorf, Germany) according to the manufacturer's instructions.

### 2.16 Statistical analysis

Experiments were performed at least three times if not indicated differently. Data were displayed as mean ± SD, and a two‐tailed Student's *t*‐test was used for statistical analysis. Pearson analysis was employed to define the correlation between two parameters, and the Kaplan–Meier method was applied for survival analysis. Data were processed using prism 9.0 (GraphPad Software, Boston, MA, USA), and statistical significance is indicated by *P*‐values: *P < 0.05; **P < 0.01; ***P < 0.001; ‘ns’ (not significant) *P* > 0.05.

## 3 Results

### 3.1 ERRFI1 is highly expressed in melanoma and associated with reduced overall survival of melanoma patients

**Fig. 1:**
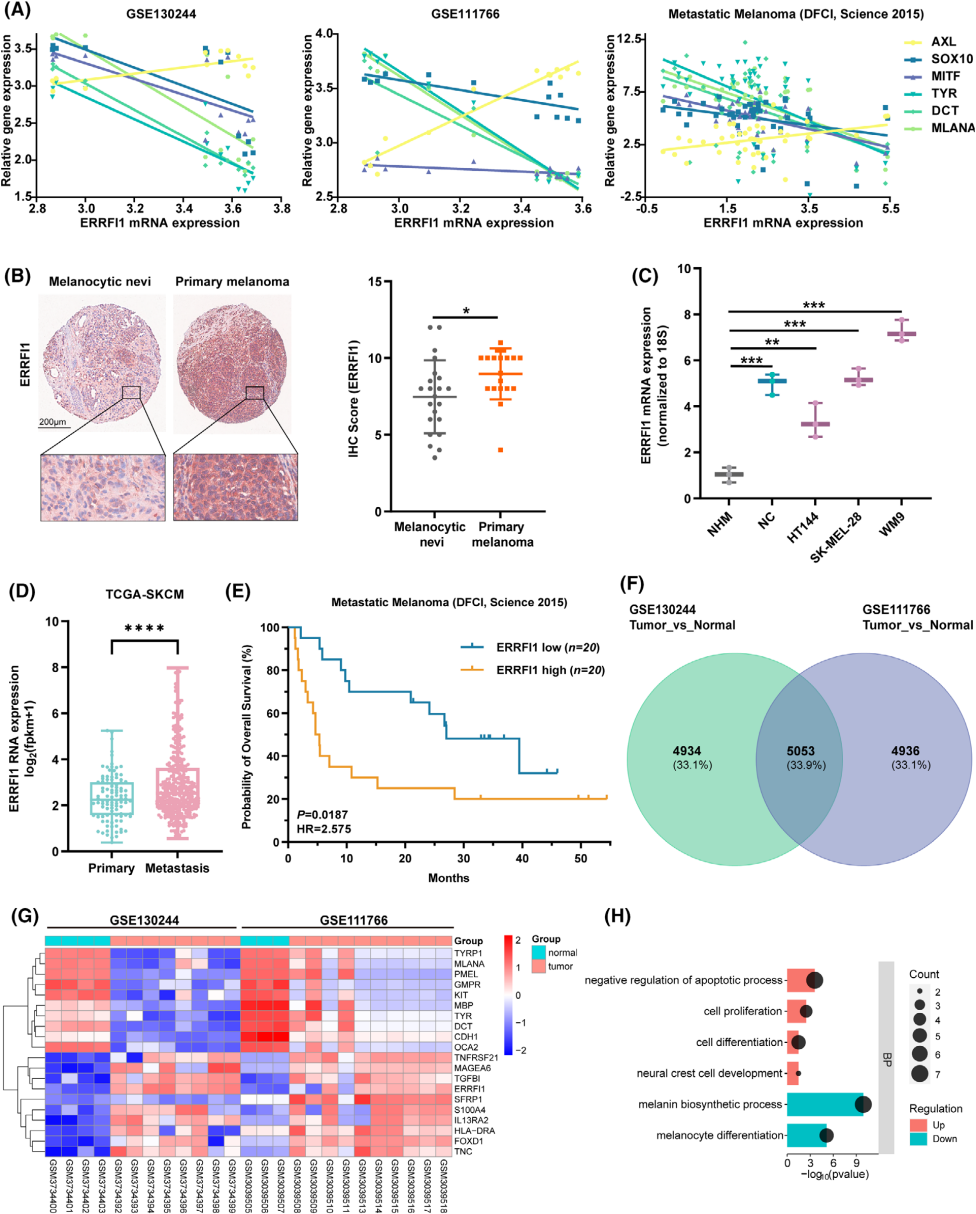
High expression levels of ERBB receptor feedback inhibitor 1 (ERRFI1) in melanoma are associated with reduced overall survival of melanoma patients. (A) ERRFI1 expression positively correlates with AXL receptor tyrosine kinase (AXL) expression and negatively with the expression of SOX10, MITF, TYR, DCT, and MLANA. The plots were generated with datasets from the GEO database (GSE130244, GSE111766) and the cBioportal database (DFCI Science 2015). Detailed information about the Pearson correlation (*r*) and the corresponding *P*‐value is provided in Fig. S1. (B) Comparison of the expression level of ERRFI1 between melanocytic nevi (*n* = 22) and primary melanoma (*n* = 19) in clinical patient samples after TMA staining (scale bar, 200 μm). Error bars depict the mean ± SD. (C) ERRFI1 mRNA expression levels in NHM, NC, and melanoma cell lines were quantified with RT‐qPCR. Data are presented as box‐and‐whisker plots showing all individual data points, whiskers indicate minimum to maximum values (*n* = 3). (D) Comparison of the ERRFI1 mRNA expression between primary (*n* = 103) and metastatic melanoma (*n* = 368). Data were taken from The Cancer Genome Atlas (TCGA) database. Data are presented as box‐and‐whisker plots showing all individual data points; whiskers indicate minimum to maximum values. Significance determined by Welch's *t*‐test, ****P < 0.0001. (E) Kaplan–Meier plot depicting the survival of melanoma patients with low or high intratumoral ERRFI1 expression. Data were taken from the cBioportal database (DFCI Science 2015). (F) Venn diagram depicting the number of overlapping genes differentially expressed between melanoma (tumor) and melanocytes (normal). Data were taken from two GSE datasets from the GEO database. (G) Heatmap generated from two GSE datasets comparing gene expression in melanoma and melanocytes and showing the top 20 dysregulated genes. (H) GO‐BP analysis of 155 genes commonly up‐ or downregulated with the threshold set to an absolute fold change of ≥ 2 and *P* < 0.05. Data were taken from the GSE130244 and GSE111766 datasets from the GEO database. Statistical significance was determined using a two‐tailed unpaired Student's *t*‐test if not stated otherwise. *P < 0.05, **P < 0.01, ***P < 0.001. BP, biological process; GO, Gene Ontology; IHC, immunohistochemistry; NC, neural crest; NHM, normal human melanocytes; SKCM, skin cutaneous melanoma; TMA, tissue microarray.

Previous studies have shown that acquired resistance to BRAFi is linked to a phenotype switch characterized by the downregulation of MITF, the master regulator of melanocyte differentiation, and the upregulation of receptor tyrosine kinases (RTKs), such as AXL, which are involved in promoting resistance to therapy [22, 23, 24, 25]. By examining datasets from three different databases, we found that ERRFI1 expression positively correlated with AXL expression but negatively with the expression of SOX10, MITF, and melanocytic differentiation markers (TYR, DCT, and MLANA) (Fig. 1A, Fig. S1A). Besides, we observed that the expression level of ERRFI1 was positively associated with the NC marker NGFR across multiple GEO datasets (Fig. S1B). Moreover, ERRFI1 displayed a significantly higher expression in primary melanomas than in melanocytic nevi (Fig. 1B). Additional comparison between melanoma cell lines (HT144, SK‐MEL‐28, and WM9) and normal human melanocytes (NHM) demonstrated that ERRFI1 levels were markedly elevated in melanoma cells compared with NHM (Fig. 1C). Analysis of data from TCGA further revealed that ERRFI1 is upregulated in metastatic melanoma relative to primary melanoma (Fig. 1D). Analysis of overall survival data from metastatic melanoma patients showed that higher intratumoral ERRFI1 expression levels were associated with decreased overall survival (Fig. 1E).

We also compared global gene expression between melanoma cells and melanocytes using data from two independent datasets (GSE130244 and GSE111766) from the GEO database and identified 5053 genes that were differentially expressed according to both datasets (Fig. 1F). Among these 5053 genes, 155 were consistently up‐ or downregulated across both datasets, with an absolute fold change of ≥ 2 and *P* < 0.05. Among the top 20 dysregulated genes, we detected ERRFI1 (Fig. 1G, Fig. S1D). Gene Ontology (GO) analysis for biological processes (BP) revealed that the upregulated genes, including ERRFI1, were predominantly associated with processes like apoptosis, cell proliferation, cell differentiation, and neural crest cell development. Conversely, downregulated genes were linked to melanin biosynthesis and melanocyte differentiation (Fig. 1H). The above results together reveal a crucial role of ERRFI1 in melanoma progression.

### 3.2 ERRFI1 KD alters the tumorigenic capacities of melanoma cells and sensitizes them to BRAFi

**Fig. 2:**
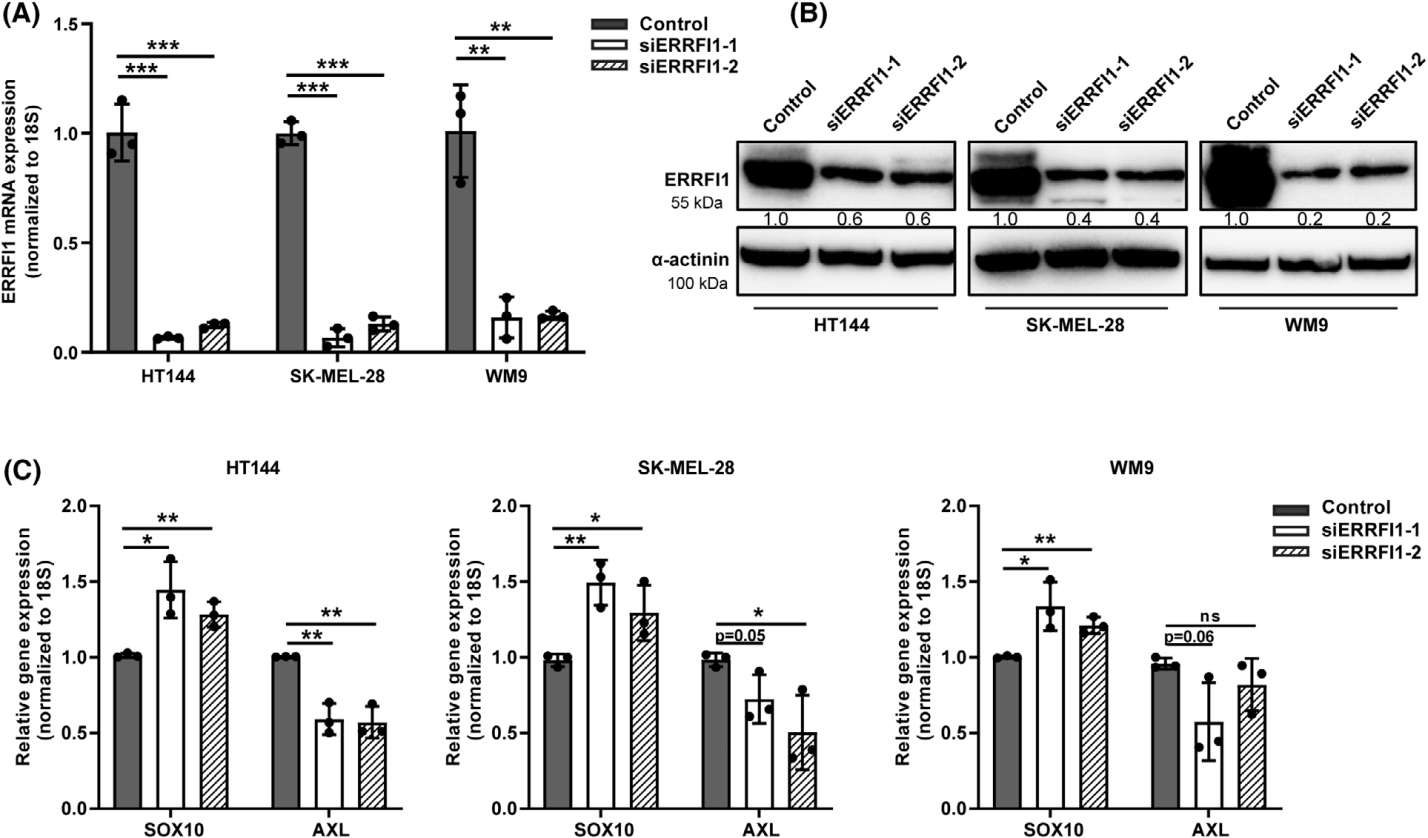
ERBB receptor feedback inhibitor 1 (ERRFI1) knockdown triggers the upregulation of SOX10 expression and the downregulation of AXL receptor tyrosine kinase (AXL) expression. (A) Validation of the successful KD of ERFFI1 in three different melanoma cell lines with the help of RT‐qPCR analysis (*n* = 3). Melanoma cells were either transfected with one of two different siRNAs targeting ERRFI1 (ERRFI1 KD) or a nontargeting siRNA (control). (B) Confirmation of the successful KD of ERRFI1 in HT144, SK‐MEL‐28, and WM9 cells with western blot. Shown are representative images from three independent experiments (*n* = 3). Melanoma cells were either transfected with one of two different siRNAs targeting ERRFI1 (ERRFI1 KD) or a nontargeting siRNA (control). The images of the α‐actinin loading controls were also used in Fig. 5G (HT144) and Fig. S5A (SK‐MEL‐28 and WM9) because the same blots were probed with multiple, different antibodies. (C) Quantification of SOX10 and AXL expression in three melanoma cell lines upon transfection with one of two different siRNAs targeting ERRFI1 (ERRFI1 KD) or a nontargeting siRNA (control). Each point represents data from three independent experiments (*n* = 3). Statistical significance was determined using a two‐tailed unpaired Student's *t*‐test. Results are shown as mean ± SD. ns, not significant; *P < 0.05, **P < 0.01, ***P < 0.001.

**Fig. 3:**
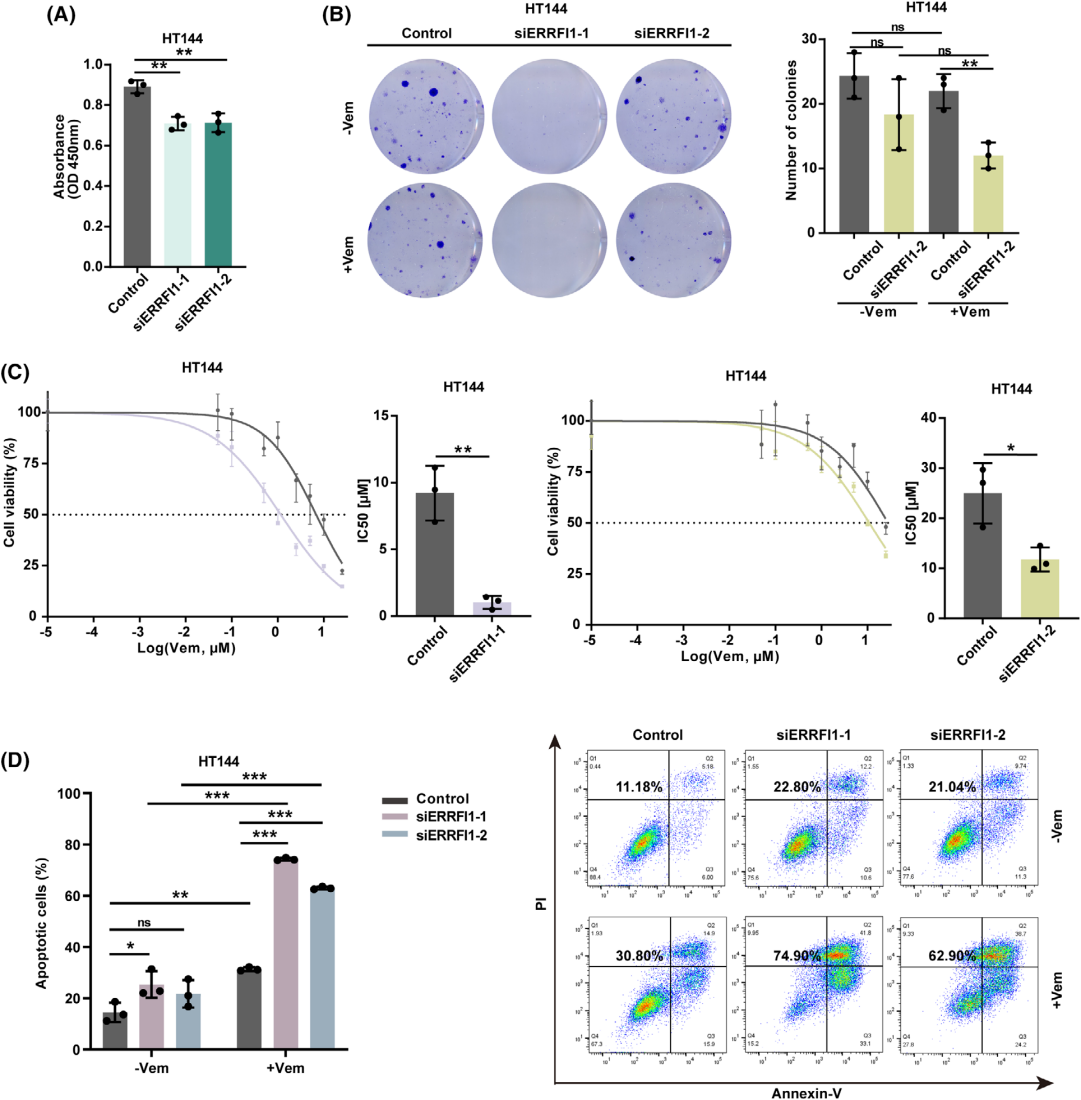
ERBB receptor feedback inhibitor 1 (ERRFI1) knockdown sensitizes melanoma cells to BRAFi. (A) BrdU ELISA proliferation assay. HT144 cells were either transfected with one of two different siRNAs targeting ERRFI1 (ERRFI1 KD) or a nontargeting siRNA (control). Forty‐eight hours later, the cells were seeded in 96‐well plates (1–2 × 10^4^ cells/well). BrdU was added for 6–24 h, and afterward, the plate was read using a TECAN Infinite M1000 PRO microplate reader set at a dual wavelength of 450/550 nm (*n* = 3). (B) Colony formation assay was performed with HT144 cells. Each well was treated with dimethylsulfoxide (DMSO) or vemurafenib (Vem) at a concentration of 10 μm for 24 h before changing the medium. Surviving cells were stained with crystal violet after 10–14 days (*n* = 3). (C) HT144 cells were either transfected with one of two different siRNAs targeting ERRFI1 (ERRFI1 KD) or a nontargeting siRNA (control). Forty‐eight hours later, the cells were seeded in a 96‐well plate (5 × 10^3^ cells/well) and then treated with vemurafenib (Vem) in a concentration range from 0.0001 to 25 μm. Cell viability was measured using the alamarBlue assay (*n* = 3). (D) HT144 cells were either transfected with one of two different siRNAs targeting ERRFI1 (ERRFI1 KD) or a nontargeting siRNA (control). Forty‐eight hours later, the cells were treated with DMSO or 10 μm vemurafenib (Vem) for another 48 h. The combined proportion of early and late apoptotic cells was calculated and displayed. Statistical analysis is shown on the left (*n* = 3). Statistical analysis was performed using Student's *t*‐test. Results are shown as mean ± SD. ns, not significant; *P < 0.05, **P < 0.01, ***P < 0.001.

To elucidate the role of ERRFI1 in melanoma progression, we employed two different siRNAs to knock down its expression in melanoma cell lines (HT144, SK‐MEL‐28, WM9). Forty‐eight hours after transfection, RT‐qPCR and western blot analysis confirmed the successful knockdown (KD) of ERRFI1 (Fig. 2A,B). Consistent with our previous findings, ERRFI1 KD led to an increase in SOX10 expression and a decrease in AXL expression in all tested cell lines (Fig. 2C). We then assessed the proliferative capacity of melanoma cells using the BrdU cell proliferation assay. Our results indicate that ERRFI1 KD cells exhibited a significantly lower proliferation rate compared with the control (Fig. 3A, Fig. S2A). Next, we performed a colony formation assay with melanoma cells transfected with ERRFI1 siRNA or a scrambled control siRNA that were additionally treated with either 10 μm dimethylsulfoxide (DMSO) or Vem for 24 h. We observed that upon treatment with Vem, the ERRFI1 KD group yielded fewer colonies compared with the control group, suggesting a role for ERRFI1 in tumorigenesis (Fig. 3B, Fig. S2B). Notably, HT144 cells transfected with siERRFI1‐1 were unable to form colonies. We conducted cell viability assays comparing the ERRFI1 KD and control groups treated with different Vem concentrations to further validate these findings. Cell viability and IC50 values were notably lower in the ERRFI1 KD group, indicating an increased sensitivity to BRAFi upon ERRFI1 KD (Fig. 3C, Fig. S2C). Additionally, apoptosis assays were performed using cells transfected with ERRFI1 siRNA, followed by treatment with DMSO or 10 μm Vem for 48 h. The apoptosis assay revealed a significantly higher proportion of apoptotic cells in the ERRFI1 KD group compared with the control group (Fig. 3D, Fig. S2D). The application of a 3D culture system provides an effective approach to linking 2D *in vitro* to *in vivo* experimental models. Here, we investigated whether ERRFI1 silencing affects the sensitivity of 3D melanoma spheroids to BRAFi treatment. ERRFI1 KD melanoma cells were first generated and subsequently induced to form 3D spheroids using a magnetic levitation approach. After spheroid stabilization, BRAFi treatment was applied for 72 h and cell viability was assessed using the alamarBlue assay. The results demonstrated that 3D melanoma spheroids derived from cells with ERRFI1 depletion exhibited lower viability compared with the control group when subjected to Vem treatment, consistent with the observations in 2D cultures (Fig. S3).

### 3.3 ERRFI1 KD resensitizes BRAFi‐resistant melanoma cells to BRAFi

**Fig. 4:**
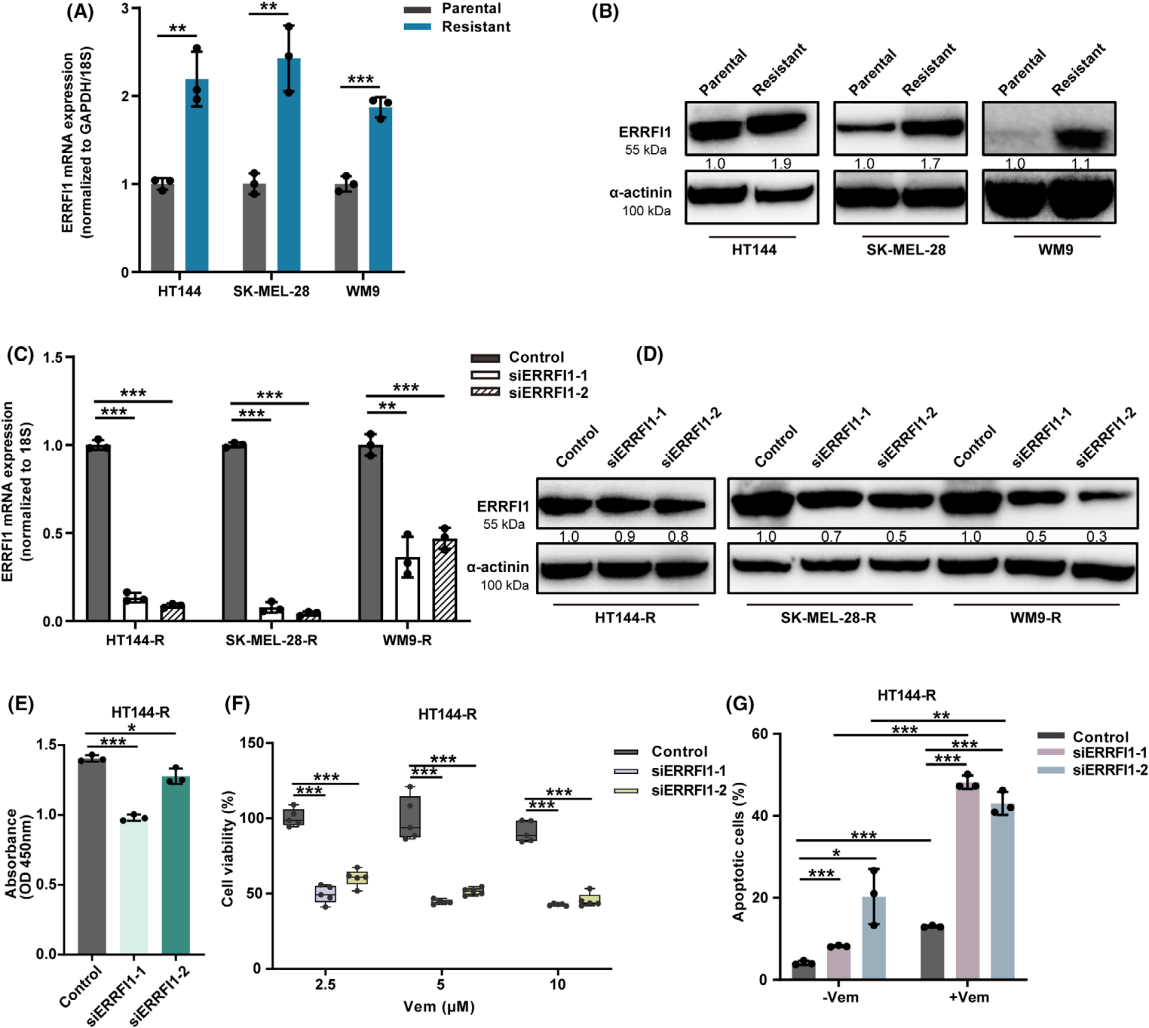
ERBB receptor feedback inhibitor 1 (ERRFI1) knockdown resensitizes BRAFi‐resistant melanoma cells to BRAFi. (A) Comparison of the ERRFI1 mRNA expression between parental and vemurafenib–resistant HT144, SK‐MEL‐28, and WM9 melanoma cells with RT‐qPCR (*n* = 3). (B) Comparison of the ERRFI1 protein expression between parental and vemurafenib–resistant HT144, SK‐MEL‐28, and WM9 cells with western blot. Depicted are representative images from three independent experiments (*n* = 3). (C) Confirmation of the successful KD of ERRFI1 in three BRAFi‐resistant melanoma cell lines with RT‐qPCR (*n* = 3). Melanoma cells were either transfected with one of two different siRNAs targeting ERRFI1 (ERRFI1 KD) or a nontargeting siRNA (control). (D) Confirmation of the successful KD of ERRFI1 in HT144‐R, SK‐MEL‐28‐R, and WM9‐R cells with western blot. Melanoma cells were either transfected with one of two different siRNAs targeting ERRFI1 (ERRFI1 KD) or a nontargeting siRNA (control). Shown are representative images from three independent experiments are shown (*n* = 3). (E) BrdU ELISA proliferation assay. HT144‐R cells were either transfected with one of two different siRNAs targeting ERRFI1 (ERRFI1 KD) or a nontargeting siRNA (control). Forty‐eight hours later, the cells were seeded in a 96‐well plate (1–2 × 10^4^ cells/well). BrdU was added for 6–24 h, and afterward, the plate was read using a TECAN Infinite M1000 PRO microplate reader set at a dual wavelength of 450/550 nm (*n* = 3). (F) HT144‐R cells were either transfected with one of two different siRNAs targeting ERRFI1 (ERRFI1 KD) or a nontargeting siRNA (control). Forty‐eight hours later, the cells were treated with different concentrations of vemurafenib (Vem) for 48 h. Cell viability was measured using the alamarBlue assay. Data are presented as box‐and‐whisker plots showing all individual data points, whiskers indicate minimum to maximum values. Shown is a representative experiment from three independent experiments with similar results (*n* = 3). (G) HT144‐R cells were either transfected with one of two different siRNAs targeting ERRFI1 (ERRFI1 KD) or a nontargeting siRNA (control). Forty‐eight hours later, the cells were treated with dimethylsulfoxide (DMSO) or 10 μm vemurafenib (Vem) for 48 h. The frequency of apoptotic cells was calculated as frequencies of Annexin V+ PI− (early apoptotic) and Annexin V+ PI+ cells (late apoptotic) within total tumor cells (*n* = 3). Statistical significance was determined using a two‐tailed unpaired Student's *t*‐test. Results are shown as mean ± SD. *P < 0.05, **P < 0.01, ***P < 0.001.

Having shown that ERRFI1 KD sensitized melanoma cells to BRAFi, we wanted to investigate the role of ERRFI1 in BRAFi‐resistant melanoma cells. For this reason, we compared mRNA and protein expression levels of ERRFI1 between BRAFi‐resistant and parental cells from the same melanoma cell line. Both levels were elevated in the resistant cells (Fig. 4A,B). Next, we knocked down ERRFI1 expression in BRAFi‐resistant cells (HT144‐R, SK‐MEL‐28‐R, WM9‐R) and confirmed the successful KD via RT‐PCR and western blot (Fig. 4C,D). Then, we performed a BrdU proliferation assay and demonstrated that ERRFI1 KD cells exhibited a reduced proliferative capacity compared with the control group, which is in accordance with the results observed for the nonresistant melanoma cells (Fig. 4E, Fig. S4A). Cell viability assays indicated an increased sensitivity of previously BRAFi‐resistant cells to BRAFi treatment (Fig. 4F, Fig. S4B). Additionally, apoptosis assays revealed higher apoptosis rates for ERRFI1 KD cells treated with 10 μm Vem compared with the control group transfected with a scrambled siRNA, corroborating that ERRFI1 KD resensitized BRAFi‐resistant melanoma cells to treatment with BRAFi (Fig. 4G, Fig. S4C).

### 3.4 Silencing ERRFI1 diminishes the activation of MAPK and AKT signaling pathways

**Fig. 5:**
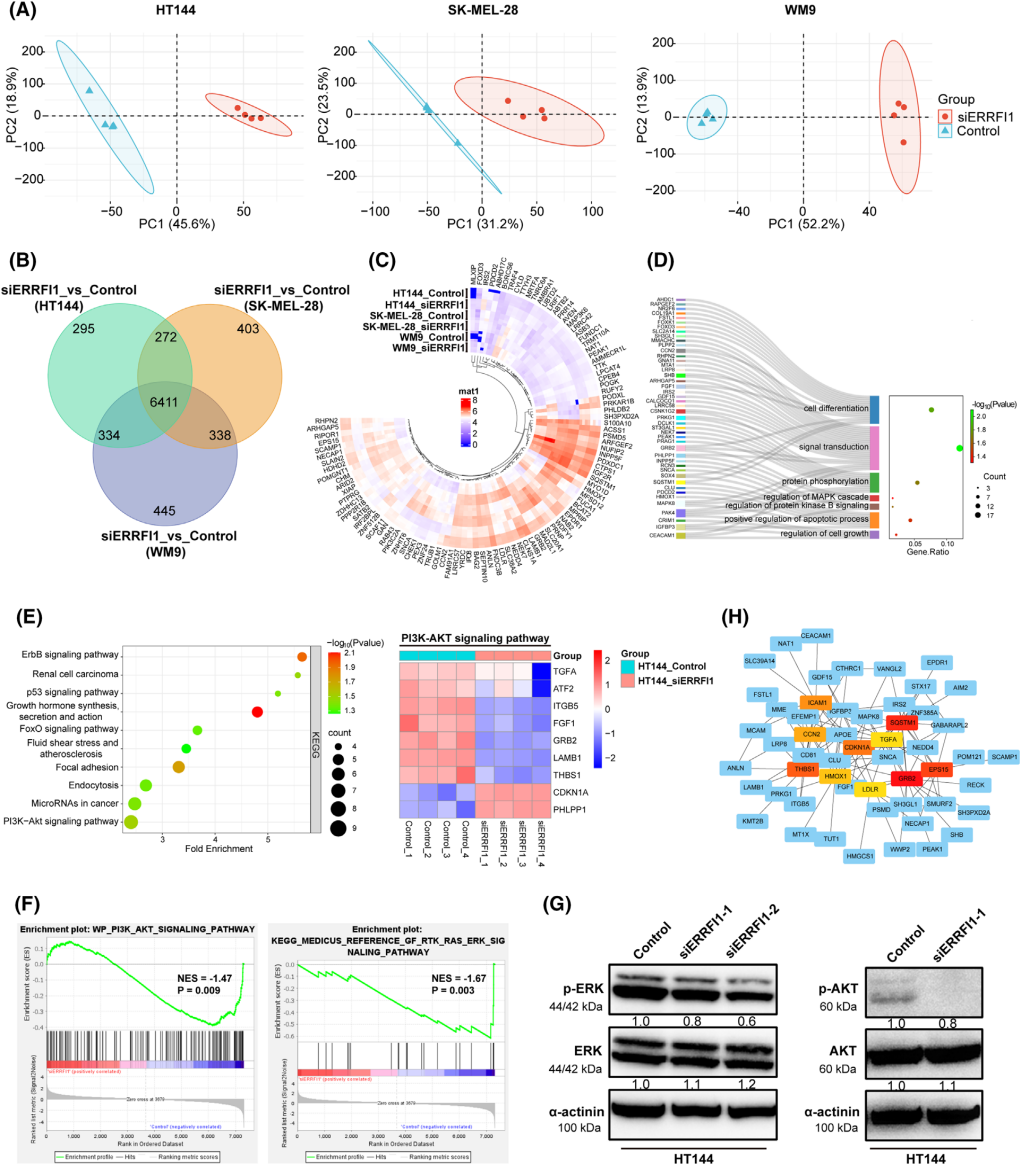
Knockdown of ERBB receptor feedback inhibitor 1 (ERRFI1) diminishes the activation of mitogen‐activated protein kinase (MAPK) and v‐akt murine thymoma viral oncogene (AKT) signaling pathways. (A) PCA results showing differences between ERRFI1 KD and control melanoma cells. Each point represents a mass spectrometry sample. Samples with similar gene expression profiles clustered together. (B) Venn diagram depicting the number of overlapping proteins differentially expressed in three melanoma cell lines. (C) Circular heatmap with hierarchical clustering displays the top 100 differentially expressed proteins (ERRFI1 KD vs Control group) across three melanoma cell lines (*P* < 0.05). Each ring corresponds to one experimental group, and each segment represents a gene. Color intensity reflects the magnitude of expression, with red indicating increased and blue decreased levels in the ERRFI1 KD group, as shown by the color scale in the center. (D) GO‐BP analysis of 183 differentially expressed proteins (FC > |1|, *P* < 0.05 in HT144 cells) highlighting their involvement in different biological processes. (E) KEGG analysis shows the top altered pathways in HT144 melanoma cells after ERRFI1 KD. The differentially expressed proteins from the KEGG PI3K‐AKT signaling pathway are shown on the right. (F) GSEA analysis of mass spectrometry data revealed that MAPK and AKT signaling pathways were significantly associated with ERRFI1. (G) Western blot analysis of the expression of components of the MAPK and AKT signaling pathways in HT144 cells either transfected with one of two different siRNAs targeting ERRFI1 (ERRFI1 KD) or a nontargeting siRNA (control). Shown are representative images from three independent experiments (*n* = 3). The images of the α‐actinin loading control in the left panel are also used in Fig. 2B because the same blots were probed with multiple, different antibodies. (H) Protein–protein interaction network analysis identified GRB2 as a central hub protein among 183 proteins that were differentially expressed between ERRFI1 KD and control melanoma cells. BP, biological process; GO, Gene Ontology; NES, normalized enrichment score; PCA, principal component analysis.

To further find the underlying molecular mechanisms of how ERRFI1 regulates the sensitization of melanoma cells to BRAFi, we compared global protein expression between the ERRFI1 KD and control groups for three melanoma cell lines (HT144, SK‐MEL‐28, and WM9) using mass spectrometry. Principal component analysis (PCA) of the resulting data demonstrated tight clustering of quadruplicate samples in each group (Fig. 5A). We identified 6411 commonly dysregulated proteins between ERRFI1 KD and control groups (Fig. 5B,C). In HT144 cells, 183 differentially expressed proteins were identified between the ERRFI1 KD and control groups based on a fold change > 1 or < −1 and a *P*‐value < 0.05. These proteins were subsequently subjected to GO enrichment analysis. GO analysis suggested that these proteins were connected to processes like cell differentiation, signal transduction, and regulation of the MAPK and AKT (PKB) pathway (Fig. 5D). KEGG pathway analysis highlighted alterations in crucial pathways, including ERBB, p53 signaling, focal adhesion, and PI3K‐AKT signaling, with key regulators, such as CDKN1A and PHLPP1 upregulated, indicating an inhibition of PI3K‐AKT signaling (Fig. 5E). Results from GSEA and western blot analysis further confirmed the reduced activation of these pathways in the HT144 ERRFI1 KD group compared with the respective control (Fig. 5F,G). Similarly, GSEA analysis based on proteomic data from SK‐MEL‐28 and WM9 cells also revealed consistent suppression of these pathways upon ERRFI1 KD (Fig. S5A,C,D). Notably, GSEA in HT144 cells also revealed a neural crest stem cell (NCSC)‐specific gene signature in the control group, whereas this enrichment was lost upon ERRFI1 KD (Fig. S5B). Interestingly, utilizing protein–protein interaction network analysis, the oncogene growth factor receptor‐bound protein 2 (GRB2) was identified as a hub protein (Fig. 5H). Studies have shown that GRB2 contributes to tumor migration, invasion, and metastasis [26, 27]. However, the relevance of the specific interaction between GRB2 and ERRFI1 for the regulation of melanoma cell resistance to BRAFi is still unclear and warrants further investigation to fully elucidate the underlying mechanisms and potential therapeutic implications.

### 3.5 miR‐200c inhibits the expression of ERRFI1 and resensitizes BRAFi‐resistant melanoma cells to BRAFi

**Fig. 6:**
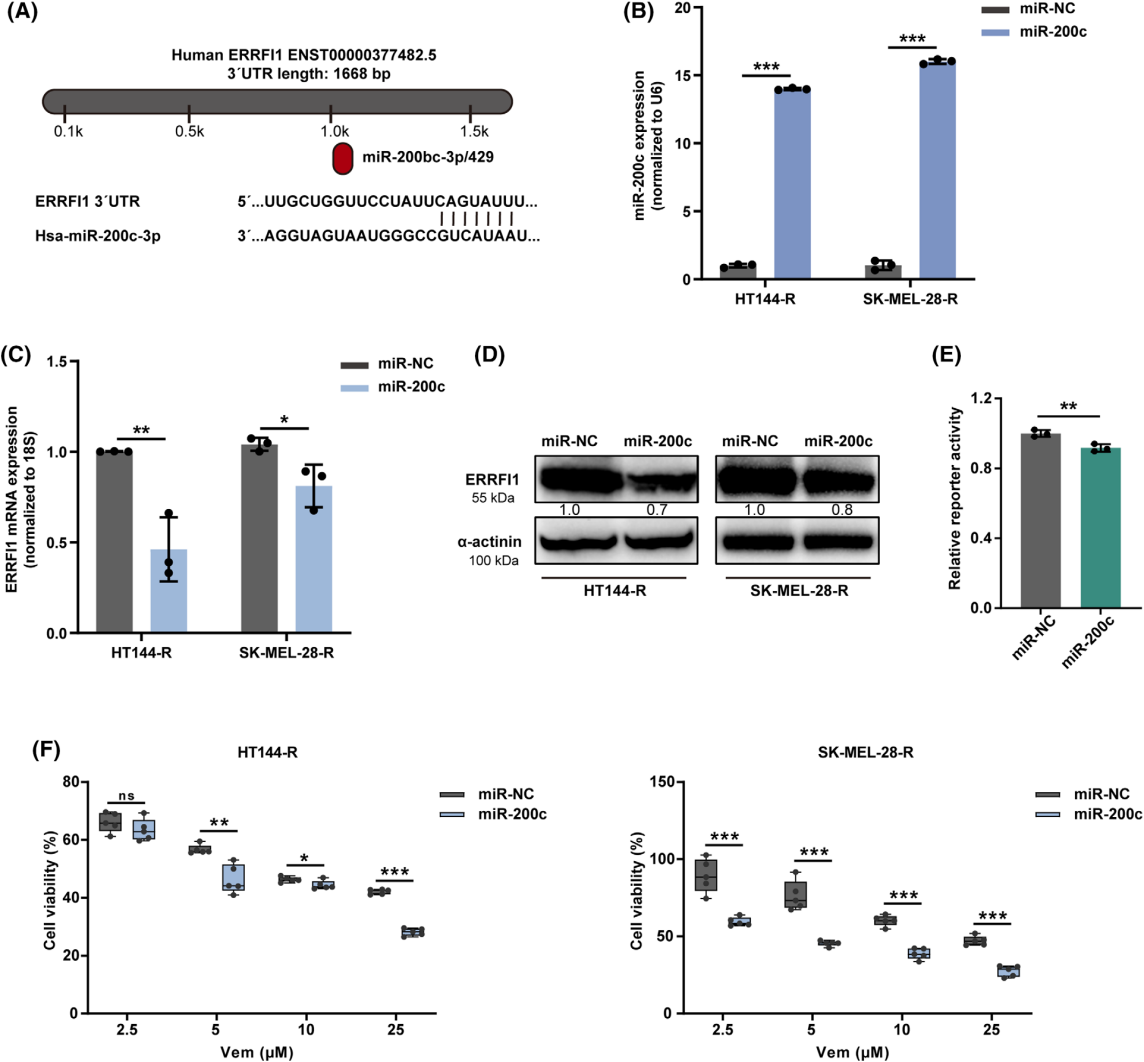
miR‐200c inhibits the expression of ERBB receptor feedback inhibitor 1 (ERRFI1) and increases the sensitivity of BRAFi‐resistant melanoma cells to BRAFi. (A) ERRFI1 was predicted as a target of miR‐200c by using TargetScan and miRDB. (B) Validation of the ectopic overexpression of miR‐200c with RT‐qPCR 56 h upon transfection of HT144‐R and SK‐MEL‐28‐R with a miR‐200c mimic or a nontargeting miRNA mimic (*n* = 3). (C) Quantification of ERRFI1 mRNA expression with RT‐qPCR in HT144‐R and SK‐MEL‐28‐R cells 56 h upon transfection with a miR‐200c mimic or a nontargeting miRNA mimic (*n* = 3). (D) Quantification of ERRFI1 protein expression with western blot in HT144‐R cells 56 h upon transfection with a miR‐200c mimic or a nontargeting miRNA mimic. Depicted are representative images from three independent experiments (*n* = 3). (E) A dual‐luciferase reporter assay was conducted in HEK293T cells to assess the direct interaction between miR‐200c and the 3′UTR (untranslated region) of ERRFI1. Cells were co‐transfected with reporter constructs containing ERRFI1 3′UTR, along with miR‐200c mimics or miR‐NC (*n* = 3). Luciferase activity was measured 48 h after transfection. (F) HT144‐R and SK‐MEL‐28‐R cells were transfected with a miR‐200c mimic or a nontargeting miRNA mimic. Fifty‐six hours later, the cells were treated with different concentrations of vemurafenib (Vem) for 72 h. Cell viability was measured using the alamarBlue assay. Data are presented as box‐and‐whisker plots showing all individual data points, whiskers indicate minimum to maximum values. Shown is a representative experiment from three independent experiments with similar results (*n* = 3). Statistical analysis was performed using a two‐tailed unpaired Student's *t*‐test. Results are shown as mean ± SD. ns, not significant; *P < 0.05, **P < 0.01, ***P < 0.001.

Increasing evidence suggests that altered expression levels of miRNAs trigger drug resistance in tumors [28, 29, 30]. For this reason, we utilized TargetScan and miRDB to identify miRNAs that could potentially bind to ERRFI1 mRNA and thereby regulate it. These tools predicted miR‐200c as a tumor‐suppressive miRNA targeting ERRFI1 (Fig. 6A). Notably, miR‐200c is downregulated in melanoma relative to nevi and has been associated with decreased drug resistance [31, 32, 33]. We transfected melanoma cells with miR‐200c mimics and analyzed the expression of ERRFI1 in response to this (Fig. 6B). The mRNA level of ERRFI1 was reduced in HT144‐R and SK‐MEL‐28‐R, and the protein level of ERRFI1 was decreased in HT144‐R upon transfection with the miR‐200c mimics (Fig. 6C,D). To evaluate whether ERRFI1 is a direct target of miR‐200c, we conducted a dual‐luciferase reporter assay using a construct containing the 3′UTR of ERRFI1. Co‐transfection with miR‐200c mimics significantly reduced luciferase activity compared with miR‐NC. The results indicate that miR‐200c directly targets the 3′UTR of ERRFI1 (Fig. 6E, Fig. S5E). Cell viability assays with BRAFi‐resistant cells treated with Vem upon transfection with miR‐200c mimics revealed an increased sensitivity of these cells to BRAFi (Fig. 6F), indicating that controlling the miR‐200c‐ERRFI1 axis could enhance the effectiveness of targeted therapy in melanoma.

## 4 Discussion

Cutaneous melanoma is a malignant tumor originating from melanocytes, which are derived from NC cells during embryonic development [34]. These NC cells originate from the ectoderm and migrate along specific pathways to their final locations, where they differentiate into various cell types, including melanocytes [35, 36]. Numerous studies have shown that genes active during NC development play a crucial role in the development of melanoma and contribute to the plasticity of melanoma cells, leading to drug resistance [37]. For instance, previous studies have shown that the NC‐associated gene MSX1 promotes melanoma development and induces its phenotypic transition [38, 39]. Heppt et al. [38] found that ectopic expression of MSX1 can induce melanoma cells to transition to an invasive and metastatic phenotype, and melanoma patients with high intratumoral MSX1 expression show lower overall survival. MITF is a well‐studied master regulator of melanogenesis that plays a key role in the transition of pluripotent NC cells to differentiated melanocytes [40]. Carreira et al. [41] demonstrated that high MITF expression is associated with a differentiated, proliferative phenotype, while low MITF expression can be connected to a stem cell‐like, invasive phenotype in melanoma. SOX10 is expressed prior to the migratory period of NC cells and plays a fundamental role in the survival of migrating NC cells and their differentiation into melanocytes [42]. Furthermore, SOX10 is integral in melanoma development and phenotype transition, and its absence enhances the stemness and invasiveness of melanoma cells [43, 44].

Additionally, our study revealed that NC‐associated genes were more highly expressed in melanoma cells compared with normal melanocytes [21, 45, 46]. These findings have important implications for melanoma treatment, suggesting that targeting these genes could improve the efficacy of existing therapies and help overcome drug resistance. For example, FOXD1, a NC‐associated gene, is upregulated in melanoma and BRAFi‐resistant melanoma cells. Knockdown of FOXD1 impairs melanoma migration and invasion and also increases the sensitivity of melanoma cells to targeted therapy [21, 45]. We also found that the downregulation of another NC‐associated gene, ID3, increases the sensitivity of melanoma cells to short‐term vemurafenib therapy [46].

Our group previously discovered that ERRFI1 is upregulated in melanoma and NC cells but not in melanocytes and that this correlates with poor prognosis [11]. This study further investigated the role of the NC‐associated gene ERRFI1, which shows elevated expression in melanoma cells, particularly those that are resistant to BRAFi (Figs 1C and 4A). Additionally, in line with our previous study, increased intratumoral ERRFI1 expression was found to correlate with a poor prognosis of melanoma patients (Fig. 1E) [11].

It has been demonstrated that the cell state characterized by MITF^low^ AXL^high^ shows an invasive phenotype and is associated with resistance to BRAFi [23, 47, 48, 49]. By analyzing datasets from the GEO and cBioportal databases, we found that ERRFI1 expression positively correlated with AXL expression but negatively with the expression of MITF, SOX10, and classic melanocytic differentiation markers, such as TYR, DCT, and MLANA (Fig. 1A). These findings provide evidence that the NC‐associated factor ERRFI1 could play an important role in regulating the dedifferentiation of melanoma cells. We further showed that the downregulation of ERRFI1 could enhance the responsiveness of melanoma cells to BRAFi (Fig. 3C). Additionally, ERRFI1 silencing restored the sensitivity of BRAFi‐resistant melanoma cells to BRAFi (Fig. 4F). These results highlight the crucial role of the NC‐associated factor ERRFI1 in modulating BRAFi resistance in melanoma cells.

Of note, BRAF‐mutant melanomas often develop resistance to BRAFi through upregulation of the MAPK and PI3K‐AKT pathways, observed in approximately 22% of patients [29, 50, 51]. Utilizing mass spectrometry‐based proteomic analysis, a list of differentially expressed proteins between ERRFI1 KD and control cells was selected, followed by GO BP enrichment analysis. As we expected, BPs, such as cell differentiation and signal transduction, were found to be affected after KD of ERRFI1 (Fig. 5D). Importantly, the downregulation of the MAPK and AKT pathways was also observed following ERRFI1 KD. By conducting KEGG pathway analysis, key factors, such as AKT‐inactivating phosphatase PHLPP1 and cyclin‐dependent kinase inhibitor 1A (CDKN1A), were found to be upregulated in ERRFI1 KD cells, underscoring the important function of ERRFI1 in regulating the AKT signaling pathway and cell proliferation (Fig. 5E). By performing GSEA analysis, we found that MAPK and AKT signaling pathways were suppressed in the ERRFI1 KD group compared with control (Fig. 5F). Consistent with the data above, decreased levels of p‐ERK and p‐AKT were investigated using western blot, indicating that ERRFI1 silencing promotes susceptibility to BRAFi by diminishing the activation of the MAPK and AKT signaling pathways (Fig. 5G).

Since ERRFI1 acted as an oncoprotein and was associated with drug resistance, we aimed at identifying an effective strategy to inhibit this factor. MicroRNAs (miRNAs) are small, noncoding RNAs that play crucial roles in the regulation of gene expression [52]. In cancer, miRNAs can function as oncogenes or tumor suppressors, influencing various cellular processes, such as proliferation, apoptosis, differentiation, and metastasis [30, 52]. Aberrant expression of miRNAs is commonly observed in cancers, where they can contribute to tumorigenesis and drug resistance [30, 53]. miR‐200c, in particular, has been shown to correlate negatively with melanoma progression and positively with patient survival [31, 54, 55]. Moreover, Liu et al. [32, 33] identified miR‐200c as a potential therapeutic agent for restoring melanoma cell sensitivity to BRAFi by targeting BMI‐1. Our findings demonstrated that miR‐200c downregulated ERRFI1 expression, which in turn resulted in the resensitization of BRAFi‐resistant melanoma cells to BRAFi treatment. However, this study lacked reverse validation from an ERRFI1 overexpression perspective and further *in vivo* experiments to strengthen the evidence. Nonetheless, these findings demonstrate promising research suggesting that targeting the miR‐200c‐ERRFI1 axis could overcome drug resistance in melanoma. This approach warrants further investigation to fully elucidate its potential as a therapeutic strategy for melanoma.

Our study identifies a novel therapeutic target for melanoma and contributes to the development of future treatment strategies. Nevertheless, several limitations should be acknowledged. An important limitation to note is that *in vivo* experiments are required to further confirm the role of ERRFI1 in melanoma progression and its negative impact on the efficacy of targeted therapy. Moreover, upon ERRFI1 KD, mass spectrometry analysis revealed suppression of the MAPK and AKT signaling pathways, and this finding was further validated by western blot. It would be more compelling to validate the pathway involvement by expressing constitutively active mutants of AKT and ERK. However, ERRFI1 KD was achieved using siRNA, which induces only a transient gene‐silencing effect. The short time window restricts the possibility of simultaneously performing lentiviral transduction to overexpress p‐AKT and p‐ERK in the context of ERRFI1 KD for further functional validation. We attempted to generate ERRFI1 KO melanoma cells using CRISPR‐Cas9 technology to investigate the role of ERRFI1 in cell growth and drug resistance. However, as demonstrated in our study, ERRFI1 is essential for melanoma cell proliferation and survival. KO of ERRFI1 led to substantial cell death and impaired cell expansion, which hindered downstream functional analyses. Optimizing experimental conditions to overcome this limitation will be important in future studies.

## 5 Conclusions

In this study, we demonstrated that ERRFI1 was highly expressed in melanoma and that its increased expression in primary tumors correlated with reduced overall survival of melanoma patients. The sensitivity of BRAFi‐resistant melanoma cells to targeted therapy could be increased by downregulating ERRFI1 expression with the help of siRNA. ERRFI1 silencing could diminish the activation of MAPK and AKT signaling pathways and thereby sensitize melanoma cells to BRAFi. In addition, miR‐200c could resensitize BRAFi‐resistant melanoma cells to BRAFi by downregulating the expression of ERRFI1. These results indicate that ERRFI1 was involved in melanoma progression and drug resistance. We conclude that ERRFI1 could be a potential therapeutic target for the treatment of melanoma.

## Conflict of interest

The authors declare no conflict of interest.

## Author contributions

NW and QS contributed to the conceptualization, methodology, formal analysis, investigation, writing—original draft, visualization, and project administration. DN, LZ, JP, TS, and YW contributed to the data analysis, data interpretation, and writing—review and editing. VU contributed to the conceptualization, supervision, data interpretation, and writing—review and editing. JU contributed to the conceptualization, supervision, data interpretation, writing—review and editing. All authors read and approved the final manuscript.

## Supporting information


**Fig. S1.** ERRFI1 is upregulated in melanoma with BRAF mutation.
**Fig. S2.** ERRFI1 KD impairs melanoma cell proliferation and increases the sensitivity of melanoma cells to BRAFi.
**Fig. S3.** Melanoma spheroids derived from ERRFI1 KD cells exhibit increased sensitivity to BRAFi.
**Fig. S4.** ERRFI1 KD resensitizes BRAFi‐resistant melanoma cells to BRAFi.
**Fig. S5.** ERRFI1 KD diminishes the activation of the MAPK and AKT signaling pathways.

## Data Availability

Supporting data related to this research are presented as Supporting Information. The original proteomics data for HT144, SK‐MEL‐28, and WM9 cells are available upon request.
