# Supplementary material for: The neural crest‐associated gene ERRFI1 is involved in melanoma progression and resistance toward targeted therapy
Source: Mol Oncol. 2025 Oct 3;20(5):1185–201. doi: 10.1002/1878-0261.70137 (PMC13155150; doi:10.1002/1878-0261.70137)
Supplement: Supplementary file 1 — Fig. S1. ERRFI1 is upregulated in melanoma with BRAF mutation. Fig. S2. ERRFI1 KD impairs melanoma cell proliferation and increases the sensitivity of melanoma cells to BRAFi. Fig. S3. Melanoma spheroids derived from ERRFI1 KD cells exhibit increased sensitivity to BRAFi. Fig. S4. ERRFI1 KD resensitizes BRAFi‐resistant melanoma cells to BRAFi. Fig. S5. ERRFI1 KD diminishes the activation of the MAPK and AKT signaling pathways. [file MOL2-20-1185-s001.zip › Supplementary information_18.09.2025.docx]

# Supplementary information

**Supplementary Figure Legends**

**Fig. S1.** ERRFI1 is upregulated in melanoma with BRAF mutation

(A) Correlation between ERRFI1 expression and the expression of AXL, SOX10, MITF, TYR, DCT, and MLANA. Detailed information about the Pearson correlation (r) and the corresponding p-value is shown. (B) Correlation between ERRFI1 expression and the expression of NGFR across GEO datasets. (C) ERRFI1 expression in BRAF- and NRAS-mutated melanomas compared to wild type (WT). (D) Comparison of the ERRFI1 mRNA expression between melanocytes (Normal) and melanoma (Tumor) from two independent datasets (GSE130244, GSE111766). Data are presented as box-and-whisker plots showing all individual data points, whiskers indicate minimum to maximum values. Statistical significance was determined using a two-tailed unpaired Student’s t-test. ^**^p<0.01, ^***^p<0.001.

**Fig. S2.** ERRFI1 KD impairs melanoma cell proliferation and increases the sensitivity of melanoma cells to BRAFi

(A) BrdU proliferation assays were conducted with SK-MEL-28 and WM9 cells. Cells were either transfected with one of two different siRNAs targeting ERRFI1 (ERRFI1 KD) or a non-targeting siRNA (control). 48h later, the cells were seeded in 96-well plates (1-2x10^4^ cells/well). BrdU was added for 6-24h, and afterwards, the plate was read using a TECAN Infinite M1000 PRO microplate reader set at a dual wavelength of 450/550 nm (n = 3). (B) Colony formation assay was performed with SK-MEL-28 cells. Each well was treated with DMSO or vemurafenib (Vem) at a concentration of 10µM for 24h before changing the medium. Surviving cells were stained with crystal violet after 10-14 days (n = 3). (C) SK-MEL-28 and WM9 cells were either transfected with one of two different siRNAs targeting ERRFI1 (ERRFI1 KD) or a non-targeting siRNA (control). 48h later, the cells were seeded in a 96-well plate (5x10^3^ cells/well) and then treated with vemurafenib (Vem) in a concentration range from 0.0001 to 25µM. Cell viability was measured using the alamarBlue assay (n = 3). (D) SK-MEL-28 cells were either transfected with one of two different siRNAs targeting ERRFI1 (ERRFI1 KD) or a non-targeting siRNA (control). 48h later, the cells were treated with DMSO or 10µM vemurafenib (Vem) for another 48h. The combined proportion of early and late apoptotic cells was calculated and displayed. Statistical analysis is shown on the left (n = 3). Statistical analysis was performed using a two-tailed unpaired Student’s t-test. Results are shown as mean ± SD. ns, not significant, ^*^p<0.05, ^**^p<0.01, ^***^p<0.001.

**Fig. S3.** Melanoma spheroids derived from ERRFI1 KD cells exhibit increased sensitivity to BRAFi

HT144 and SK-MEL-28 cells were seeded in a 3D culture 96-well plate after ERRFI1 KD and treated with DMSO or 10µM vemurafenib (Vem) for 72h upon spheroid formation. Cell viability was measured using the alamarBlue assay. The left panel displays representative micrographs of HT144 and SK-MEL-28 cells grown as 3D spheroids (scale bar: 200 µm). The right panel depicts cell viability in ERRFI1 KD and control spheroids with or without Vem treatment (n = 3). Statistical analysis was performed using a two-tailed unpaired Student’s t-test. Results are shown as mean ± SD. ns, not significant, ^**^p<0.01, ^***^p<0.001.

**Fig. S4.** ERRFI1 KD resensitizes BRAFi-resistant melanoma cells to BRAFi

(A) BrdU proliferation assays were performed in SK-MEL-28-R and WM9-R cells. Cells were either transfected with one of two different siRNAs targeting ERRFI1 (ERRFI1 KD) or a non-targeting siRNA (control). 48h later, the cells were seeded in a 96-well plate (1-2x10^4^ cells/well). BrdU was added for 6-24h, and afterwards, the plate was read using a TECAN Infinite M1000 PRO microplate reader set at a dual wavelength of 450/550 nm (n = 3). (B) SK-MEL-28-R and WM9-R cells were either transfected with one of two different siRNAs targeting ERRFI1 (ERRFI1 KD) or a non-targeting siRNA (control). 48h later, the cells were treated with different concentrations of vemurafenib (Vem) for 48h. Cell viability was measured using the alamarBlue assay. Data are presented as box-and-whisker plots showing all individual data points, whiskers indicate minimum to maximum values.Shown is a representative experiment from three independent experiments with similar results (n = 3). (C) SK-MEL-28-R and WM9-R cells were either transfected with one of two different siRNAs targeting ERRFI1 (ERRFI1 KD) or a non-targeting siRNA (control). 48h later, the cells were treated with DMSO or 10µM vemurafenib (Vem) for 48h. The proportion of apoptotic cells was measured using an annexin V assay. The combined proportion of early and late apoptotic cells was calculated (n = 3). Statistical significance was determined using a two-tailed unpaired Student´s t-test. Results are shown as mean ± SD. ns, not significant, ^*^p<0.05, ^**^p<0.01, ^***^p<0.001.

**Fig. S5.** ERRFI1 KD diminishes the activation of the MAPK and AKT signaling pathways

(A) Western blot analysis of the expression of components of the MAPK and AKT signaling pathway in SK-MEL-28 and WM9 cells either transfected with one of two different siRNAs targeting ERRFI1 (ERRFI1 KD) or a non-targeting siRNA (control). Shown are representative images from three independent experiments (n = 3). The images of the α-actinin loading controls were also used in Figure 2B because the same blots were probed with multiple, different antibodies. (B) GSEA of mass spectrometry data in HT144 cells identified NCSC-specific gene signatures in control and ERRFI1 KD groups. (C) GSEA plot showing significant impairment of the MAPK signaling pathway in SK-MEL-28 cells upon ERRFI1 KD. (D) GSEA plots showing reduced activity of the PI3K-AKT signaling pathway and the ERK signaling pathway in WM9 cells upon ERRFI1 KD. (E) A dual-luciferase reporter assay was conducted in HEK293T cells to assess the direct interaction between miR-200c and the 3´UTR of ERRFI1. Cells were co-transfected with a empty control vector, along with miR-200c mimics or miR-NC (n=3). Luciferase activity was measured 48h after transfection. Statistical significance was determined using a two-tailed unpaired Student’s t-test. Results are shown as mean ± SD. ns, not significant.
