# Supplementary figures and images for: The neural crest‐associated gene ERRFI1 is involved in melanoma progression and resistance toward targeted therapy
Source: Mol Oncol. 2025 Oct 3;20(5):1185–201. doi: 10.1002/1878-0261.70137 (PMC13155150; doi:10.1002/1878-0261.70137)

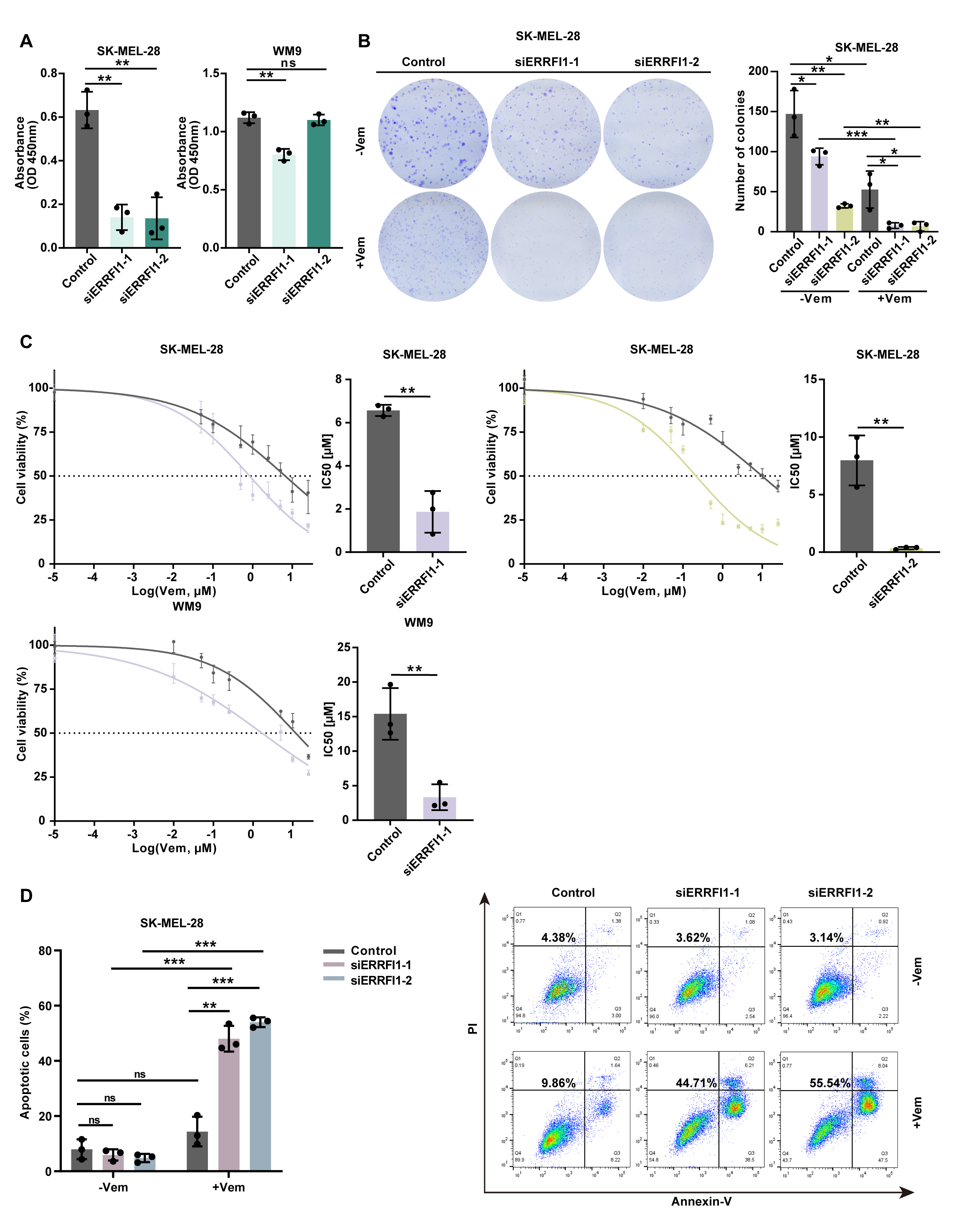

Supplement: Supplementary file 1 — Fig. S1. ERRFI1 is upregulated in melanoma with BRAF mutation. Fig. S2. ERRFI1 KD impairs melanoma cell proliferation and increases the sensitivity of melanoma cells to BRAFi. Fig. S3. Melanoma spheroids derived from ERRFI1 KD cells exhibit increased sensitivity to BRAFi. Fig. S4. ERRFI1 KD resensitizes BRAFi‐resistant melanoma cells to BRAFi. Fig. S5. ERRFI1 KD diminishes the activation of the MAPK and AKT signaling pathways. [file MOL2-20-1185-s001.zip › mol270137-sup-0002-FigureS2.tif]

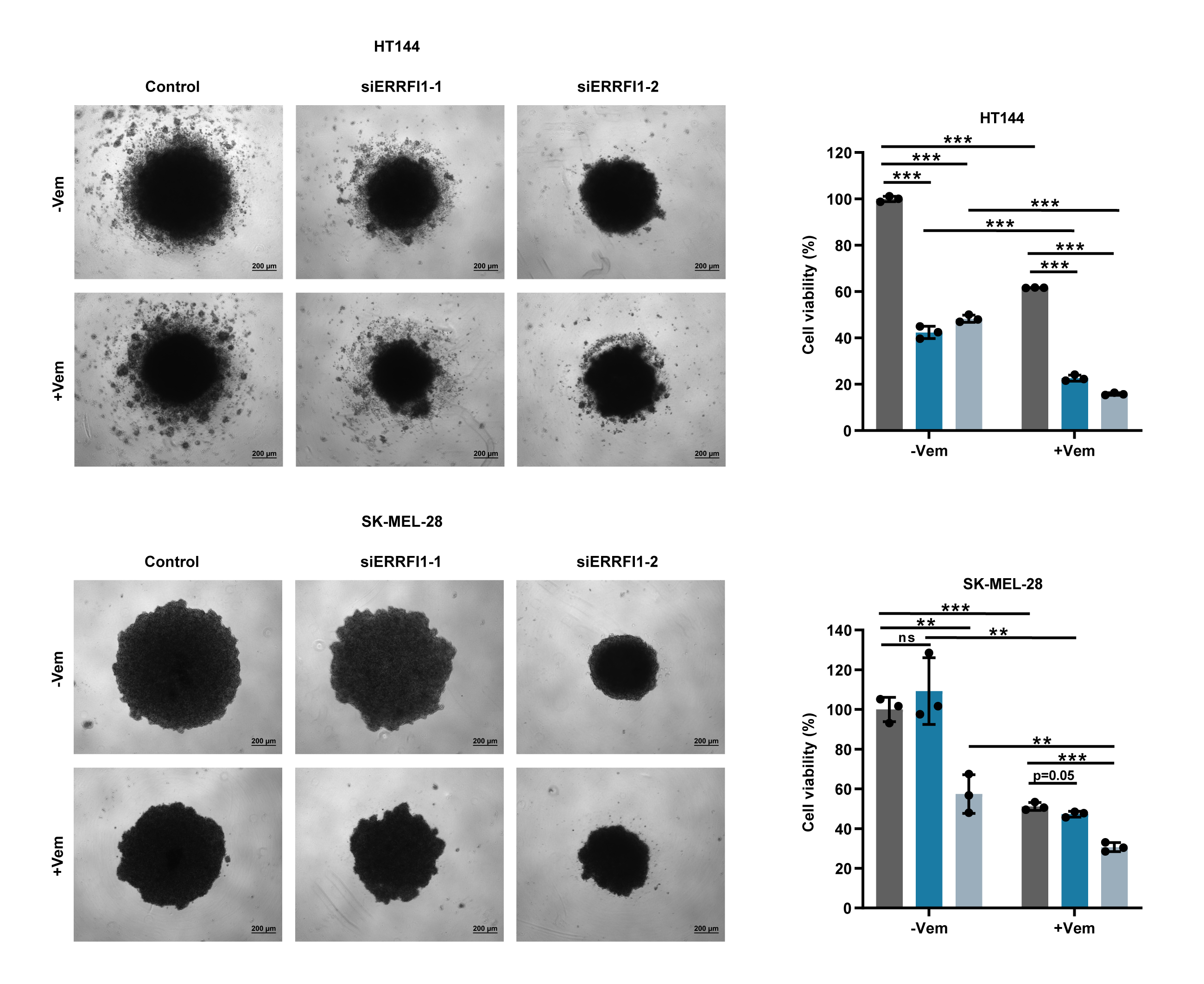

Supplement: Supplementary file 1 — Fig. S1. ERRFI1 is upregulated in melanoma with BRAF mutation. Fig. S2. ERRFI1 KD impairs melanoma cell proliferation and increases the sensitivity of melanoma cells to BRAFi. Fig. S3. Melanoma spheroids derived from ERRFI1 KD cells exhibit increased sensitivity to BRAFi. Fig. S4. ERRFI1 KD resensitizes BRAFi‐resistant melanoma cells to BRAFi. Fig. S5. ERRFI1 KD diminishes the activation of the MAPK and AKT signaling pathways. [file MOL2-20-1185-s001.zip › mol270137-sup-0003-FigureS3.tif]

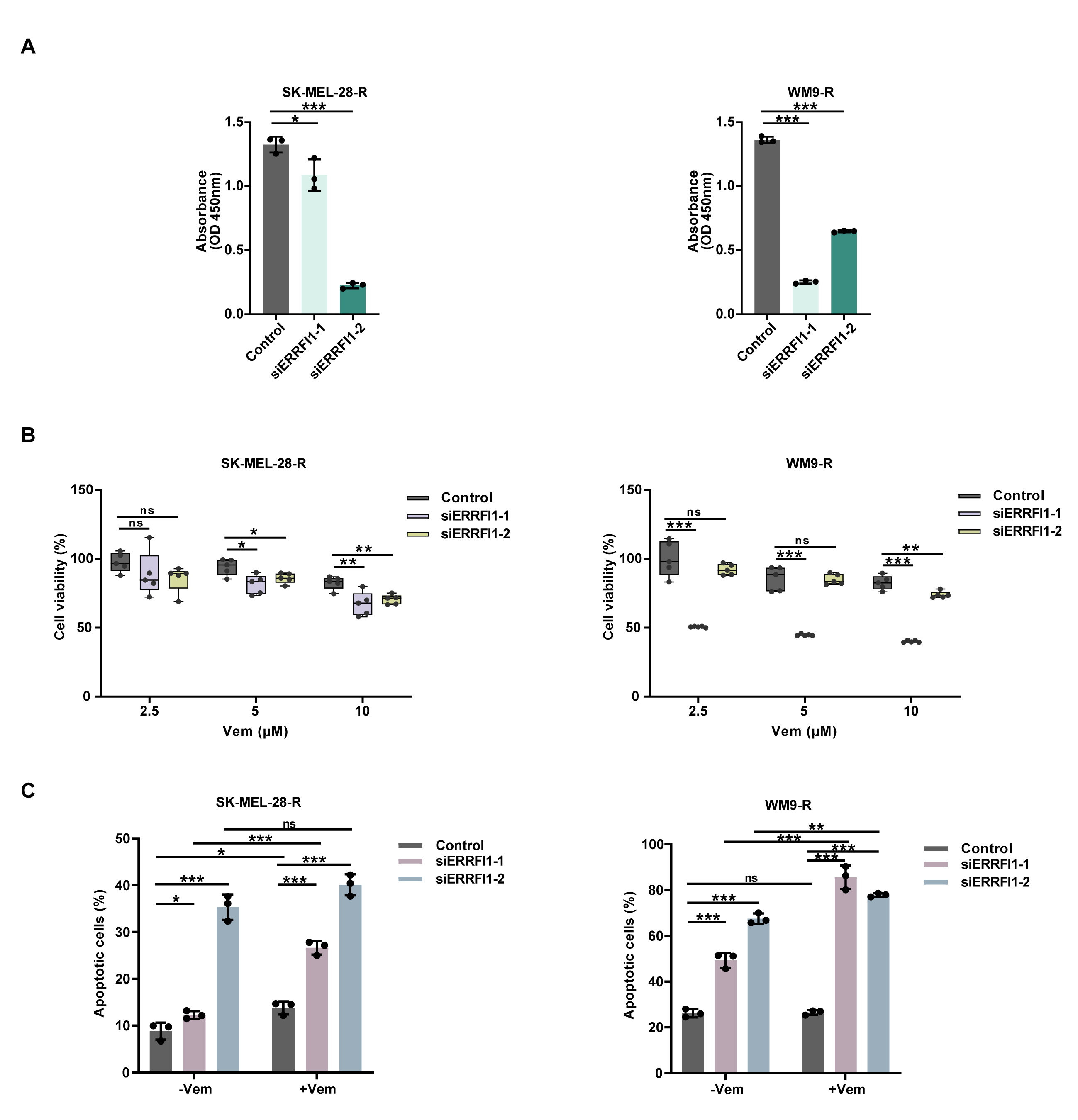

Supplement: Supplementary file 1 — Fig. S1. ERRFI1 is upregulated in melanoma with BRAF mutation. Fig. S2. ERRFI1 KD impairs melanoma cell proliferation and increases the sensitivity of melanoma cells to BRAFi. Fig. S3. Melanoma spheroids derived from ERRFI1 KD cells exhibit increased sensitivity to BRAFi. Fig. S4. ERRFI1 KD resensitizes BRAFi‐resistant melanoma cells to BRAFi. Fig. S5. ERRFI1 KD diminishes the activation of the MAPK and AKT signaling pathways. [file MOL2-20-1185-s001.zip › mol270137-sup-0004-FigureS4.tif]

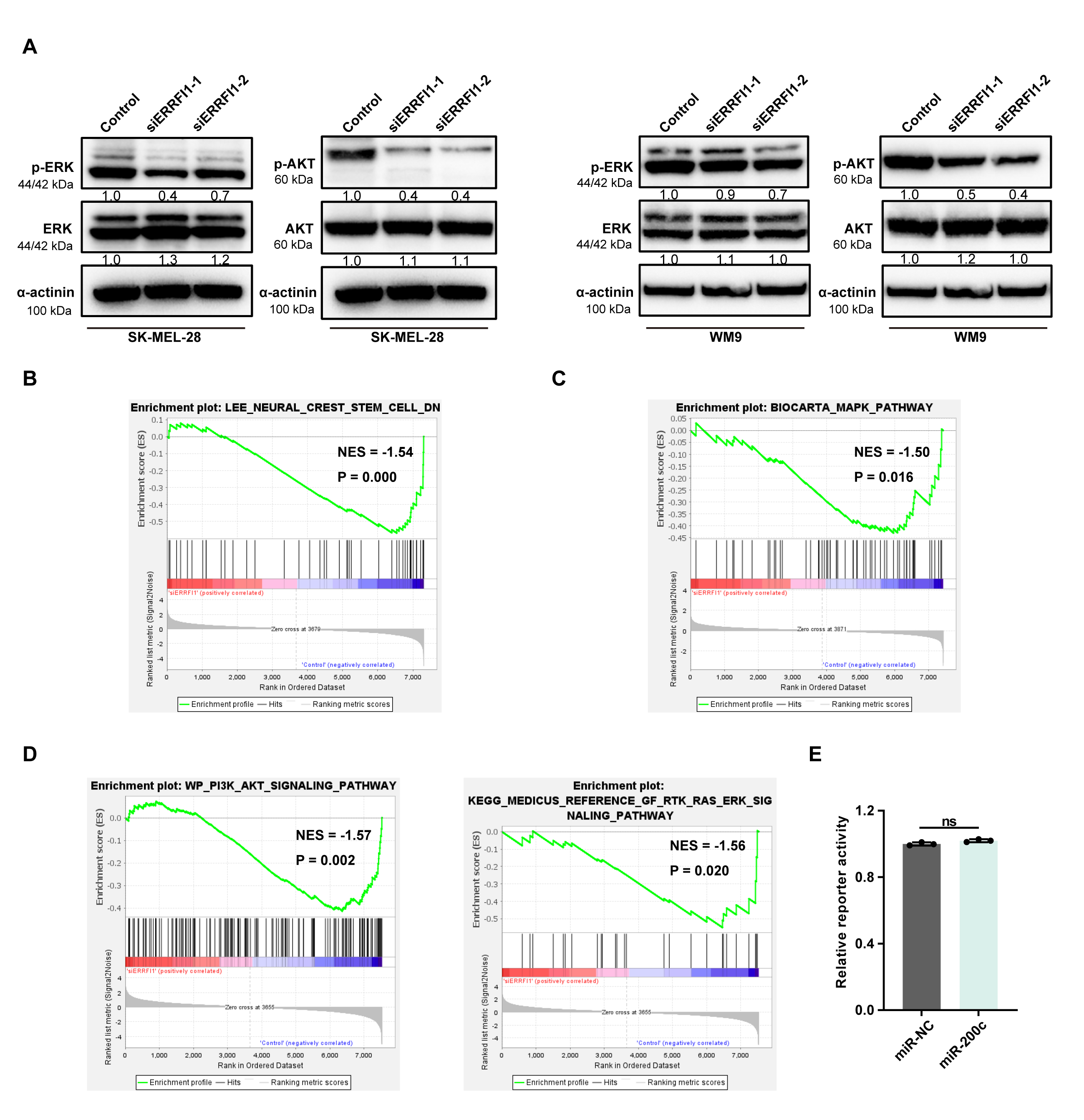

Supplement: Supplementary file 1 — Fig. S1. ERRFI1 is upregulated in melanoma with BRAF mutation. Fig. S2. ERRFI1 KD impairs melanoma cell proliferation and increases the sensitivity of melanoma cells to BRAFi. Fig. S3. Melanoma spheroids derived from ERRFI1 KD cells exhibit increased sensitivity to BRAFi. Fig. S4. ERRFI1 KD resensitizes BRAFi‐resistant melanoma cells to BRAFi. Fig. S5. ERRFI1 KD diminishes the activation of the MAPK and AKT signaling pathways. [file MOL2-20-1185-s001.zip › mol270137-sup-0005-FigureS5.tif]

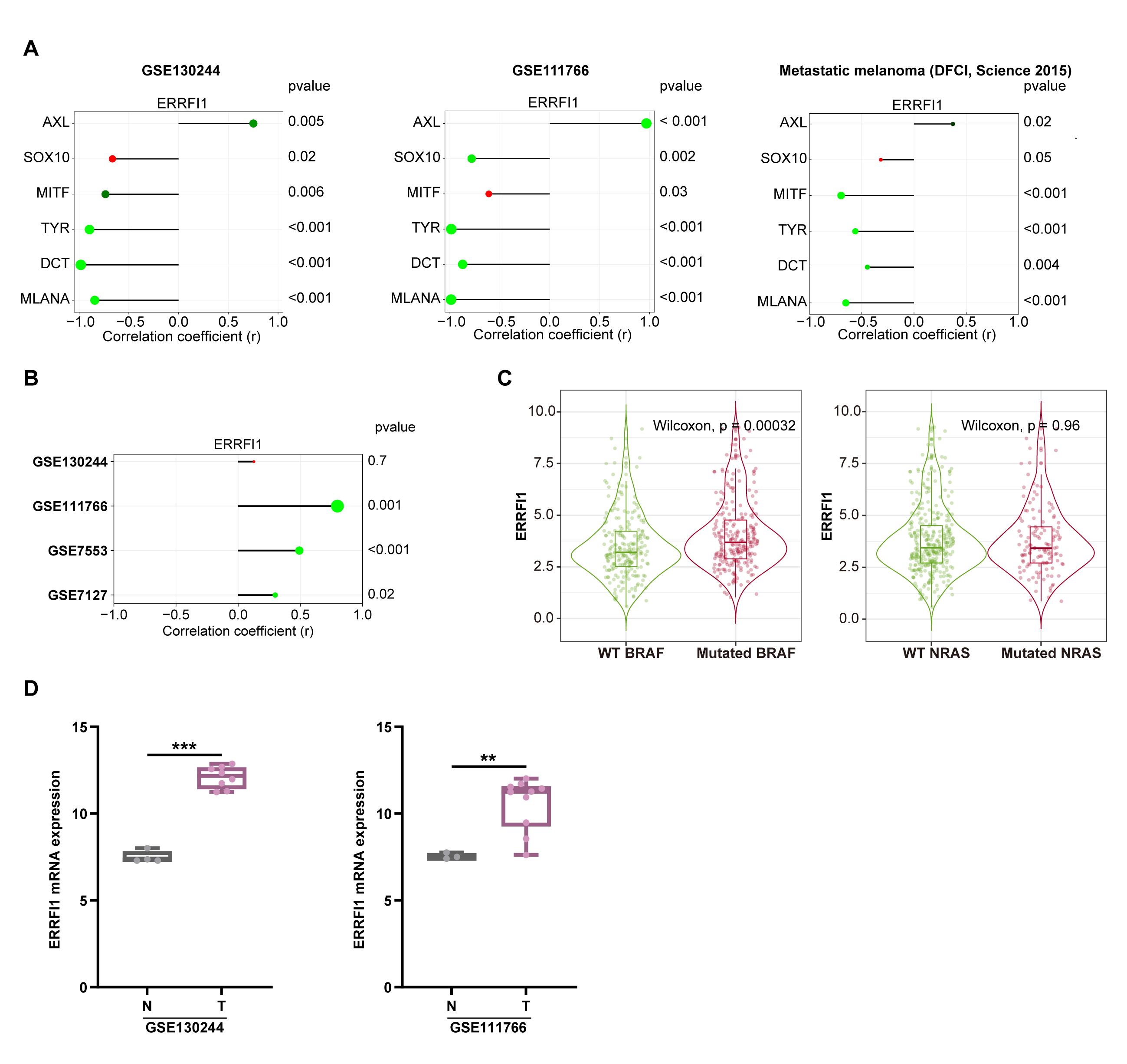

Supplement: Supplementary file 1 — Fig. S1. ERRFI1 is upregulated in melanoma with BRAF mutation. Fig. S2. ERRFI1 KD impairs melanoma cell proliferation and increases the sensitivity of melanoma cells to BRAFi. Fig. S3. Melanoma spheroids derived from ERRFI1 KD cells exhibit increased sensitivity to BRAFi. Fig. S4. ERRFI1 KD resensitizes BRAFi‐resistant melanoma cells to BRAFi. Fig. S5. ERRFI1 KD diminishes the activation of the MAPK and AKT signaling pathways. [file MOL2-20-1185-s001.zip › mol270137-sup-0001-FigureS1.tif]
